# Conserved function of bat TBK1 in activating innate immunity against RNA viruses: insights into the innate immune response in bats

**DOI:** 10.3389/fimmu.2025.1574866

**Published:** 2025-07-28

**Authors:** Qiuju Liu, Qi Shao, Caixia Xu, Chao Liu, Shuhan Li, Jie Wang, Xia Liu, Kehui Zhang, Yaxian Yan, Jianhe Sun, Yuqiang Cheng

**Affiliations:** ^1^ Shanghai Key Laboratory of Veterinary Biotechnology, School of Agriculture and Biology, Shanghai Jiao Tong University, Shanghai, China; ^2^ Key Laboratory of Veterinary Pathobiology and Disease Control, College of Animal Science and Technology, Anhui Agricultural University, Hefei, Anhui, China; ^3^ Shandong Provincial Key Laboratory of Zoonoses, Sino-German Cooperative Research Centre for Zoonosis of Animal Origin of Shandong Province, College of Veterinary Medicine, Shandong Agricultural University, Shandong, China

**Keywords:** BAT, TBK1, virus, innate immunity, IFNβ

## Abstract

Bats exhibit unique abilities to coexist with viruses asymptomatically, setting them apart among mammals. The innate immune system serves as the primary defense against pathogens. As a crucial central node protein in this system, TANK binding kinase 1(TBK1) can receive signals from multiple pattern recognition receptors (PRRs), and then promote the production of Type I interferon (IFN I) and inflammatory factors. Despite its importance, how TBK1 works in bats remains poorly understood. Here, through bioinformatics analysis, TBK1 was found to exhibit a high sequence conservation across species. Overexpression of bat TBK1 significantly upregulated IFNβ expression, and then inhibited viral replication. Co-expression of bat TBK1 with bat IRF1/3/7 can facilitate the upregulation of IFNβ mediated by bat TBK1, implying the activation signals potentially can be transmitted from bat TBK1 to IRF1/3/7, and then promote IFNβ production. Structurally, protein kinase domain (PKD), ubiquitin-like domain (ULD), and coiled-coil domain 1 (CCD1) are essential domains for bat TBK1 to function normally. In summary, this study elucidated bat TBK1 has a conserved ability to activate bat antiviral innate immunity.

## 1 Introduction

Bats are distinctive among mammals for their capability to fly (1), employ laryngeal echolocation (2), and their unusual tendency to host a wide variety of viruses. More than 4,100 viruses can infect bats. Many species of bats (e.g., rhinolophids, hipposiderids, pteropodids) have been shown to tolerate and survive many viruses that have high mortality rates in humans (3–5), such as SARS-CoV-2, MERS-Cov, Marburg, and henipaviruses (6–10). Also, bat genomes contain a high diversity of ancient and contemporary viral insertions (11, 12), suggesting that bats have a long and tolerant evolutionary history with their viral pathogens.

The innate immune system serves as the primary defense against pathogens. Pattern recognition receptors(PRRs) recognize pathogen-associated molecular patterns (PAMPs) of pathogenic microorganisms, thereby initiating the innate immune response. Through cascading signal transduction, downstream transcription factors such as NF-κB and interferon regulatory factors (IRFs) are activated, leading to the production of inflammatory cytokines and type I interferons(IFNα/β) to defend against pathogenic microorganisms. Bats’ innate immune system exhibits a series of unique features differing from other mammals. For example, IFNα is inductively expressed in humans, but constitutively expressed in bats (13), which enhanced host defence responses; A highly conserved residue of STING mutated in bat, caused STING-dependent type I IFN response was dampened (14). This helps avoid abnormally excessive activation of bats’ innate immune system during the antiviral process. It’s potentially due to these unique features of bat innate immune response rendering bat as asymptomatic and tolerant viral hosts (13).

In mammals, TANK binding kinase 1(TBK1) is a crucial central node protein in the innate immune system. TBK1 can receive activation signals from multiple PRRs such as toll-like receptors (TLRs), RIG-I-like receptors (RLRs), Nod-like receptor (NLRs), and cytoplasmic DNA receptors (15). It can be activated by signaling pathways including TLR3/4-TRIF, RIG-I-MAVS, and cGAS-cGAMP-STING, which subsequently phosphorylate downstream transcription factors IRFs and NF-κB. The phosphorylated NF-κB and IRFs then translocate to the nucleus, promoting the transcription of type I interferons and inflammatory cytokines. However, how TBK1 works in bats is still poorly understood. Considering the importance of TBK1 in innate immunity and the uniqueness of bat innate immune system, it is significant to elucidate the function of TBK1 in bats and to determine whether it has unique characteristics in bats.

Here, we first investigated the response of bat TBK1 to viral invasion. After vesicular stomatitis virus (VSV) infection, bat TBK1 expression level was up-regulated. Subsequently, the *Tadarida brasiliensis* TBK1 was cloned, and bioinformatics analysis revealed that TBK1 exhibits a high conservation across species. This suggests that TBK1’s function is possible conserved among species. The dual luciferase reporter assay and quantitative real-time PCR showed that overexpression of bat TBK1 significantly upregulated IFNβ expression, and then inhibited viral replication. Further, bat TBK1 related innate immune signaling pathways were preliminarily studied. Co-expression of bat TBK1 with bat IRF1/3/7 can facilitate the upregulation of IFNβ mediated by bat TBK1, implying the activation signals potentially can be transmitted from bat TBK1 to IRF1/3/7, and then promote IFNβ production. The essential domain of bat TBK1 for antiviral innate immunity function was also elucidated. Protein kinase domain (PKD), ubiquitin-like domain (ULD), and coiled-coil domain 1 (CCD1) are essential domains for bat TBK1 to promote IFNβ production.

## 2 Materials and methods

### 2.1 Cell culture

Human embryonic kidney cell line (293T), chicken embryonic fibroblast cell line (DF1), and bat lung cell line (TB1LU) were procured from ATCC. TB1LU was isolated in 1965 from the lung of adult bat(*Tadarida brasiliensis*). All cell were maintained in DMEM supplemented with 10% FBS and incubated at 37°C in a 5% CO^2^ environment.

### 2.2 virus infection

Newcastle disease virus (NDV) strain was obtained from the China Institute of Veterinary Drug Control (Beijing, China). The vesicular stomatitis virus (VSV) stored in our Laboratory. The calculation of virus TCID50 is performed using the Reed-Muench method. One day prior to viral infection, cells were seeded into cell culture plates at 4 × 10^5^/well of 24-well plate or 8 × 10^5^/well of 12-well plate. When the cell density reaches approximately 90%, the virus is diluted to 0.1 MOI using DMEM (Without FBS). Then, discard the medium from the cell culture plate, rinse three times with PBS, and add DMEM containing the virus at 0.1 MOI.

### 2.3 Cloning of bat TBK1

Based on the Molossus molossus TBK1 sequence (XM_036252234.1) obtained from the National Center for Biotechnology Information (NCBI), the primers bat TBK1-F and bat TBK1-R (**Supplementary Table 1**) were designed and used to clone bat TBK1 from TB1Lu cells cDNA. The PCR product was ligated into a pTOPO-Blunt vector (Vazyme Biotech Co., ltd) and sent to the Beijing Genomics Institute (Beijing, China) for sequencing. Primer design and sequence alignment were performed using SnapGene.

### 2.4 Plasmid construction

Construction of pcDNA3.1-bat-TBK1: The pcDNA3.1-bat-TBK1 plasmids were generated by integrating the full-length bat TBK1 gene into the linearized pcDNA3.1 expression vector via homologous recombination. The pcDNA3.1 vector was digested with the restriction enzymes EcoRV and HindIII. Construction of pcDNA3.1-bat-IRF1, pcDNA3.1-bat-IRF3 and pcDNA3.1-bat-IRF7: Using the cDNA of TB1LU as a template, bat IRF1, bat IRF3 and bat IRF7 were cloned. Under the action of homologous recombinase, the full-length bat IRF1, bat IRF3 and bat IRF7 were respectively ligated into the linearized pcDNA3.1, constructing the plasmids pcDNA3.1-bat-IRF1, pcDNA3.1-bat-IRF3 and pcDNA3.1-bat-IRF7. Construction of pcDNA3.1-bat-TBK1-dPKD, pcDNA3.1-bat-TBK1-dULD, and pcDNA3.1-bat-TBK1-dCCD1: The deletion mutant plasmids of bat TBK1, namely pcDNA3.1-bat-TBK1-dPKD, pcDNA3.1-bat-TBK1-dULD, and pcDNA3.1-bat-TBK1-dCCD1, which lack the PKD, ULD, or CCD1 domains, respectively, were constructed by inverse PCR using the pcDNA3.1-bat-TBK1 plasmid as a template. The chicken IFN-β promoter luciferase reporter plasmids (pGL-chIFN-β-Luc), which contained −158 to +14 of the chIFN-β promotor motif, were constructed as described in our previous study (16). The human IFN-β promoter luciferase reporter plasmids (pGL-huIFN-β-Luc) was stored in our laboratory. Plasmid transformation was carried out using DH5α Chemically Competent Cells (Tsingke Biotechnology, Beijing, China). The primers used in all plasmid construction process are listed in **Supplementary Table 1**.

### 2.5 Plasmid transfection

Cells were seeded in 12-well or 24-well plates (NEST Biotechnology, Wuxi, China) at 3 × 10^5^/well of 24-well plate or 6 × 10^5^/well of 12-well plate one day before transfection. And the plasmid was transfected 250 ng/well in 24-well or 500 ng/well in 12-well. Transfection procedure follows protocol of Nulen Plus-Trans™ Transfection Reagent (Nulen, Shanghai, China). The ratio of transfection reagent to plasmid was 1.5 µL of transfection reagent per 500 ng of plasmid in 293Tcells and 1uL of transfection reagent per 500ng of plasmid in DF1 and TB1LU cells.

### 2.6 Luciferase reporter assay

The DF-1 and 293T cells were plated in 24-well plates, after confluence reached 80%, cells were transiently co-transfected with 1) the aim plasmid pcDNA3.1-bat-TBK1 plasmids or truncated plasmids of bat TBK1 (250 ng/well), 2) the reporter plasmid PGL-IFNβ-Luc (120 ng/well) and 3) the control Renilla luciferase (PRL-TK, 60 ng/well). The cells were lysed 24 hours after transfection, and luciferase activity was detected.

### 2.7 RNA extraction and quantitative Real-Time PCR

Cells’ total RNAs were extracted with AG RNAex Pro Reagent and then was reverse-transcribed to cDNA. Operation method references the instructions provided by the kit (Vazyme), SYBR green PCR mix was used as fluorescent dye(Vazyme) and the cDNA was analyzed with the Roche LightCycler 96. Relative quantitative analysis of gene expression levels was conducted using the 2^−ΔΔCt^ method. The β-actin was the internal reference when examining the level of genes. The primer sequences for the genes are shown in the **Supplementary Table 1**.

### 2.8 Western blot analysis

The cells’ total proteins were extracted, and then separated via SDS-PAGE. Then the protein is transferred from the PAGE to the membrane. The membrane was incubated in primary antibody including ant-FLAG (Yeasen) and β-tubulin at 4°C overnight. After washing off the primary antibody, the secondary antibody was added for 1 h incubation at 4°C shaker. Then wash off the secondary antibodies and place the membrane into the developer for about one minute. After development, imaging was conducted using the Tanon 5200 imaging system. (Tanon, Shanghai, China).

### 2.9 Statistical analysis

The results were expressed as the mean ± standard deviation (SD). GraphPad Prism 8.0 was employed for graphical representation of the data. Data were analyzed by using the student’s t-test, one-way ANOVA or two-way ANOVA. P < 0.05 indicates statistical significance, P < 0.01 denotes highly significant differences. In figures, * indicates P<0.05, ** indicates P<0.01, *** indicates P<0.001, **** indicates P<0.0001. In this study, all bar graphs have been normalized relative to the first bar in each respective graph.

## 3 Results

### 3.1 Upregulation of bat TBK1 expression in response to VSV infection.

**Figure 1:**
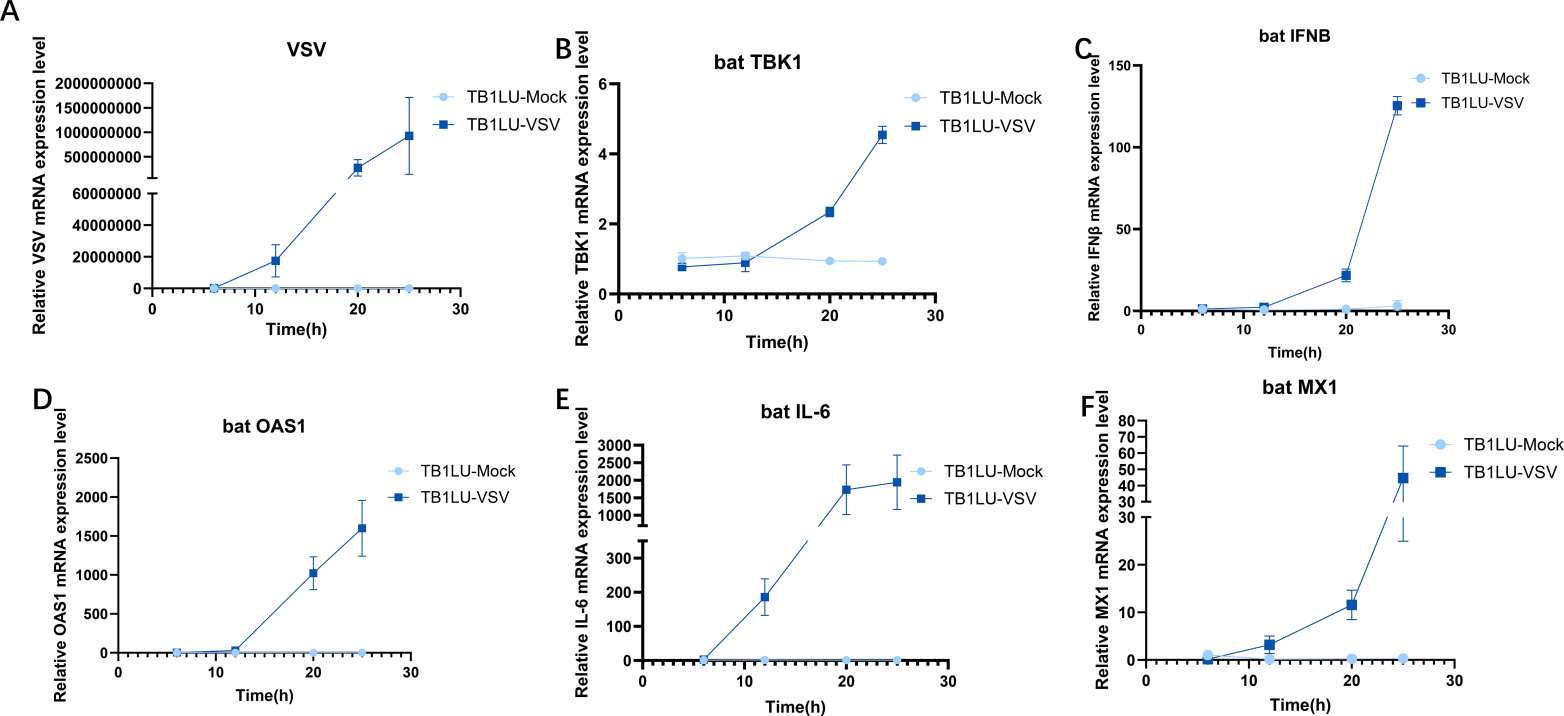
Upregulation of bat TBK1 expression in response to VSV infection. RT-qPCR was used to detect the expression level of VSV, bat TBK1, bat IFNβ, bat OAS1, bat IL-6 and bat MX1 in TB1Lu cells infection with VSV at 0.1MOI. **(A)** Replication of VSV at 6, 12, 20, 25h of infection. **(B)** Expression of TBK1 at 6, 12, 20, 25h of VSV infection. **(C-F)** Expression of IFNβ, OAS1, IL6, MX1 at 6, 12, 20, 25h of VSV infection. In this study, All relative quantitative analysis of gene expression levels was conducted using the 2^−ΔΔCt^ method. The β-actin was the internal reference when examining the level of genes. The primer sequences for the genes are shown in the **Supplementary Table 1**. During data normalization, the 6-hour data of the uninfected group was used as the reference.

The response of bat TBK1 to virus invasion is unclear. To elucidate this, TB1LU was infected with VSV at 0.1 MOI, after infection for 6, 12, 20, 25h, expression of TBK1, VSV, IFNβ, OAS1, IL6, MX1 were detected by quantitative real-time PCR. During data normalization, the 6-hour data of the uninfected group was used as the reference. The results showed that replication of VSV increased gradually with the longer duration of infection (**Figure 1A**). TBK1, IFN, OAS1, IL6, MX1 expression levels were up-regulated after VSV infection. (**Figures 1B-F**) The up-regulation of bat TBK1 expression after VSV infection suggests that bat TBK1 may be involved in resistance to virus invasion.

### 3.2 Bioinformatic analysis of bat TBK1

**Figure 2:**
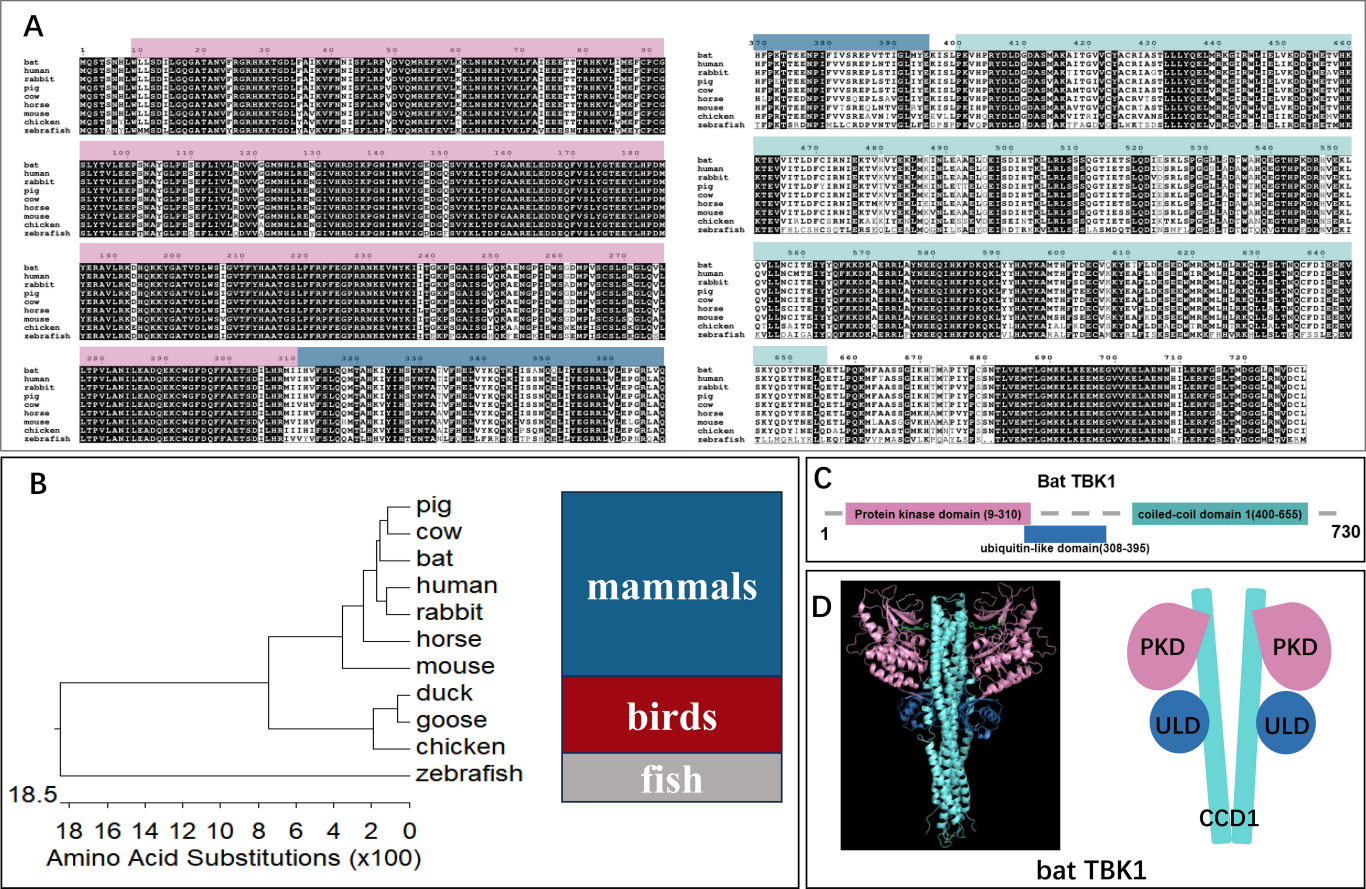
The bioinformatics analysis of bat TBK1. **(A)** The amino acid sequence of bat TBK1 was aligned with other species, including human (NP_037386.1), rabbit (XP_051701601.1), pig (NP_001098762.1), cow (NP_001179684.1), horse (XP_023499698.1), mouse (NP_062760.3), chicken (NP_001186487.1), and zebrafish (NP_001038213.2) using Clustal W and edited with ESPript 3.0. **(B)** A phylogenetic tree was constructed based on the TBK1 from different species, including mammals (pig, cow, bat, human, rabbit, horse, mouse), birds (duck, goose, chicken), fish (zebrafish). **(C)** The functional domains of bat TBK1 were predicted using InterPro. **(D)** Three-dimensional structure of bat TBK1 was predicted by Swiss Model and edited by PyMOL. The pink area, blue area and light blue area are protein kinase domain (PKD), ubiquitin-like domain (ULD), and coiled-coil domain 1 (CCD1) respectively.

To elucidate the function of TBK1 in antiviral process, we cloned *Tadarida brasiliensis* TBK1 using the *Tadarida brasiliensis* 1 lung (TB1Lu) cell line cDNA and conducted bioinformatic analysis. First, the amino acid sequence of bat TBK1 was aligned with other species, including human(NP_037386.1), rabbit(XP_051701601.1), pig(NP_001098762.1), cow(NP_001179684.1), horse(XP_023499698.1), mouse(NP_062760.3), chicken(NP_001186487.1), and zebrafish(NP_001038213.2) (**Figure 2A**). The results demonstrated high sequence conservation of TBK1 across species, suggesting that bat TBK1 may possess functions similar to TBK1 in other species. To further clarify evolutionary relationships between bat TBK1 and TBK1 from other species, we constructed phylogenetic tree of TBK1, which is composed of three branches: mammals, birds, and fish. Bat TBK1 is most closely to pig TBK1 and cattle TBK1 in evolution (**Figure 2B**). Returning to the analysis of bat TBK1, the functional domains of bat TBK1 were predicted using InterPro. Bat TBK1 is composed of protein kinase domain (PKD: AA9-310), ubiquitin-like domain(ULD: AA308-395) and coiled-coil domain 1(CCD1:AA400-655) (**Figure 2C**). In structural composition, bat TBK1 conforms to the characteristics of the IKK family. Three-dimensional structure of bat TBK1 was predicted by Swiss Model and edited by PyMOL. (**Figure 2D**) The extensive interactions between PKD, ULD, and CCD1 form a compact TBK1 dimer.

### 3.3 Function of bat TBK1 in antiviral innate immunity

**Figure 3:**
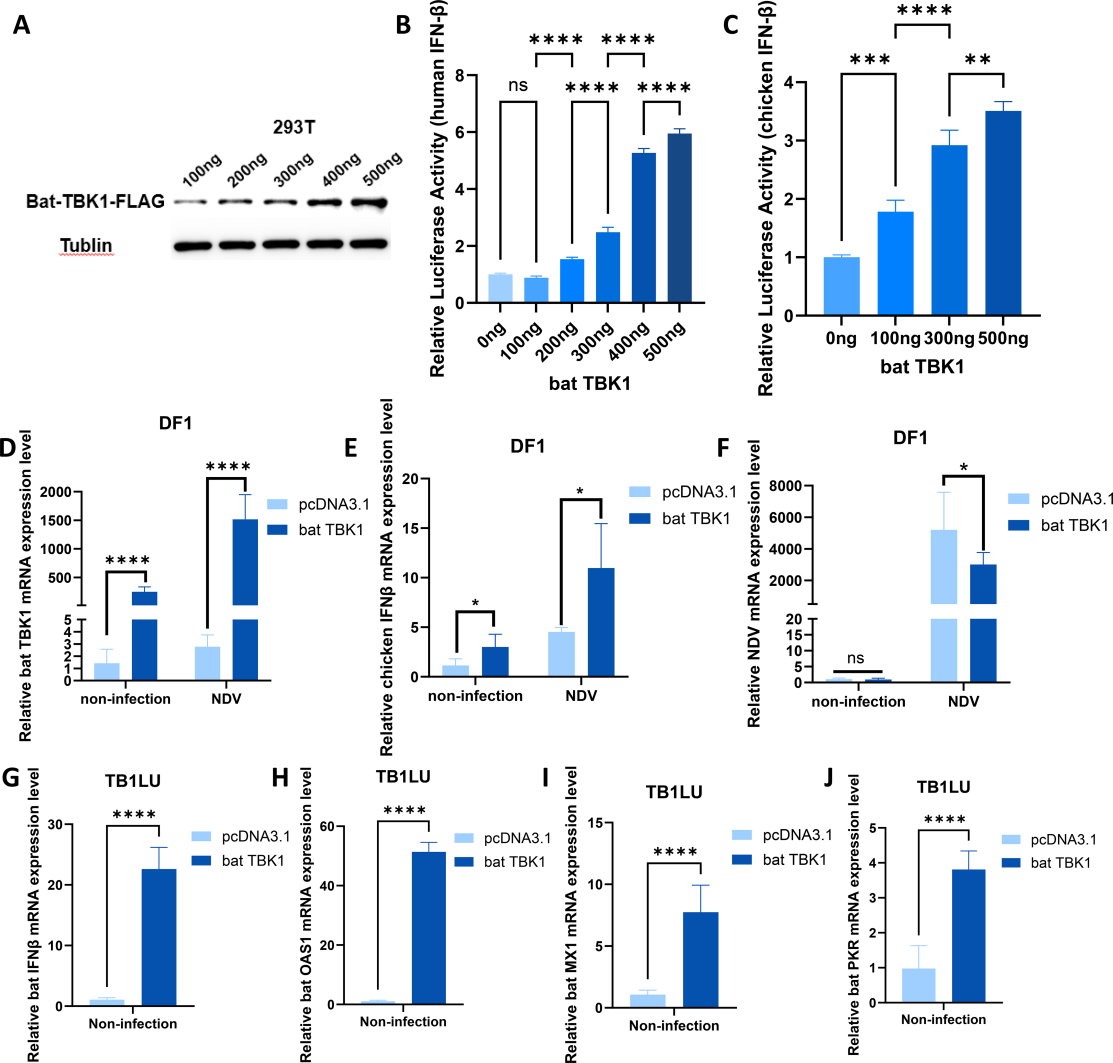
Function of bat TBK1 in antiviral innate immunity. **(A)** pcDNA3.1-bat-TBK1-FLAG was transfected into 293T cells at 100ng or 200ng or 300ng or 400ng or 500ng. At 24 h post-transfection, cellular proteins were harvested for Western blot analysis to examine TBK1 expression. The C-terminus of bat TBK1 was conjugated with a FLAG tag, and the expression level of bat TBK1 was detected using a FLAG antibody, with Tublin serving as the internal reference. **(B)** 0ng, 100ng, 200ng, 300ng, 400ng, or 500ng of pcDNA3.1-bat-TBK1 was co-transfected with 120ng of PGL-human IFNβ-Luc and 60ng of PRL-TK into 293T cells (The total amount of transfected plasmids was maintained at 680 ng, with the remaining portion supplemented by empty pcDNA3.1 vector). At 24 hours post-transfection, cells were lysed, and dual-luciferase reporter assays were performed to quantify IFNβ expression. The results indicated that overexpression of bat TBK1 significantly enhanced IFNβ expression in a dose-dependent manner. When the transfection amount of pcDNA3.1-bat-TBK1 is 100 ng, the quantity is too low to promote IFNB expression, or the promoting effect is too minimal to be detected. Data were analyzed by using the one-way ANOVA. **(C)** 0ng, 100ng, 300ng, or 500ng of pcDNA3.1-bat-TBK1 was co-transfected with 120ng of PGL-chicken IFNβ-Luc and 60ng of PRL-TK into DF1 cells (The total amount of transfected plasmids was maintained at 680 ng, with the remaining portion supplemented by empty pcDNA3.1 vector). At 24 hours post-transfection, cells were lysed, and dual-luciferase reporter assays were performed to quantify IFNβ expression. Data were analyzed by using the one-way ANOVA. **(D–F)** DF1 cells were transfected with 500 ng of either pcDNA3.1-bat-TBK1 or empty pcDNA3.1 vector. Six hours post-transfection, the cells were infected with NDV at 0.1 MOI. After 24 hours of infection, total RNA was extracted and reverse-transcribed into cDNA, followed by qPCR analysis to measure the mRNA expression levels of bat TBK1, chicken IFNβ, and NDV. Data were analyzed by using the two-way ANOVA. **(G–J)** TB1LU cells were transfected with 500 ng of either pcDNA3.1-bat-TBK1 or empty pcDNA3.1 vector. After 24 hours of infection, total RNA was extracted and reverse-transcribed into cDNA, followed by qPCR analysis to measure the mRNA expression levels of bat IFNβ, bat OAS1, bat MX1 and bat PKR. Data were analyzed by using the student’s t-test. In this figure, * indicates P<0.05, ** indicates P<0.01, *** indicates P<0.001, **** indicates P<0.0001, ns indicates “not significant” (p > 0.05), all bar graphs have been normalized relative to the first bar in each respective graph and the sequence of all qPCR primers are shown in **Supplementary Table 1**.

The high similarity of TBK1 sequences between bats and other species suggests that bat TBK1 potentially possess functions similar to other species. To clarify this, We constructed a bat TBK1 overexpression plasmid: pcDNA3.1-bat-TBK1. Western blot analysis confirmed that transfection of this plasmid into cells can upregulate the expression of bat TBK1 protein (**Figure 3A**). Bats are the only mammals capable of sustained flight, exhibiting convergent evolution with birds in aerodynamic adaptations, despite their distinct evolutionary origins. Therefore, this study of bat TBK1 utilized multiple cell lines including the TB1LU cells (representative of bats), along with 293T cells (representative of other mammals) and DF1 cells (representative of birds). Different doses of pcDNA3.1-bat-TBK1 was co-transfected with 120ng of PGL-IFNβ-Luc and 60ng of PRL-TK into 293T or DF1 cells. At 24 hours post-transfection, cells were lysed, and dual-luciferase reporter assays were performed to quantify IFNβ expression. The results indicated that overexpression of bat TBK1 significantly enhanced IFNβ expression in a dose-dependent manner (**Figures 3B, C**). At the transcriptional level, qPCR analysis revealed that overexpression of TBK1 during NDV infection enhances IFNβ expression and inhibits NDV viral replication in DF1 cells (**Figures 3D-F**). To definitively characterize the function of bat TBK1, experiments in bat cell lines are indispensable. Similar to other species’ cell lines, overexpression of bat TBK1 in the TB1LU cell line also significantly enhanced the production of IFNβ as well as interferon-stimulated genes (ISGs) including OAS1, MX1, and PKR (**Figures 3G-J**).

### 3.4 Bat TBK1 promotes IFNβ production through bat IRF1/3/7

**Figure 4:**
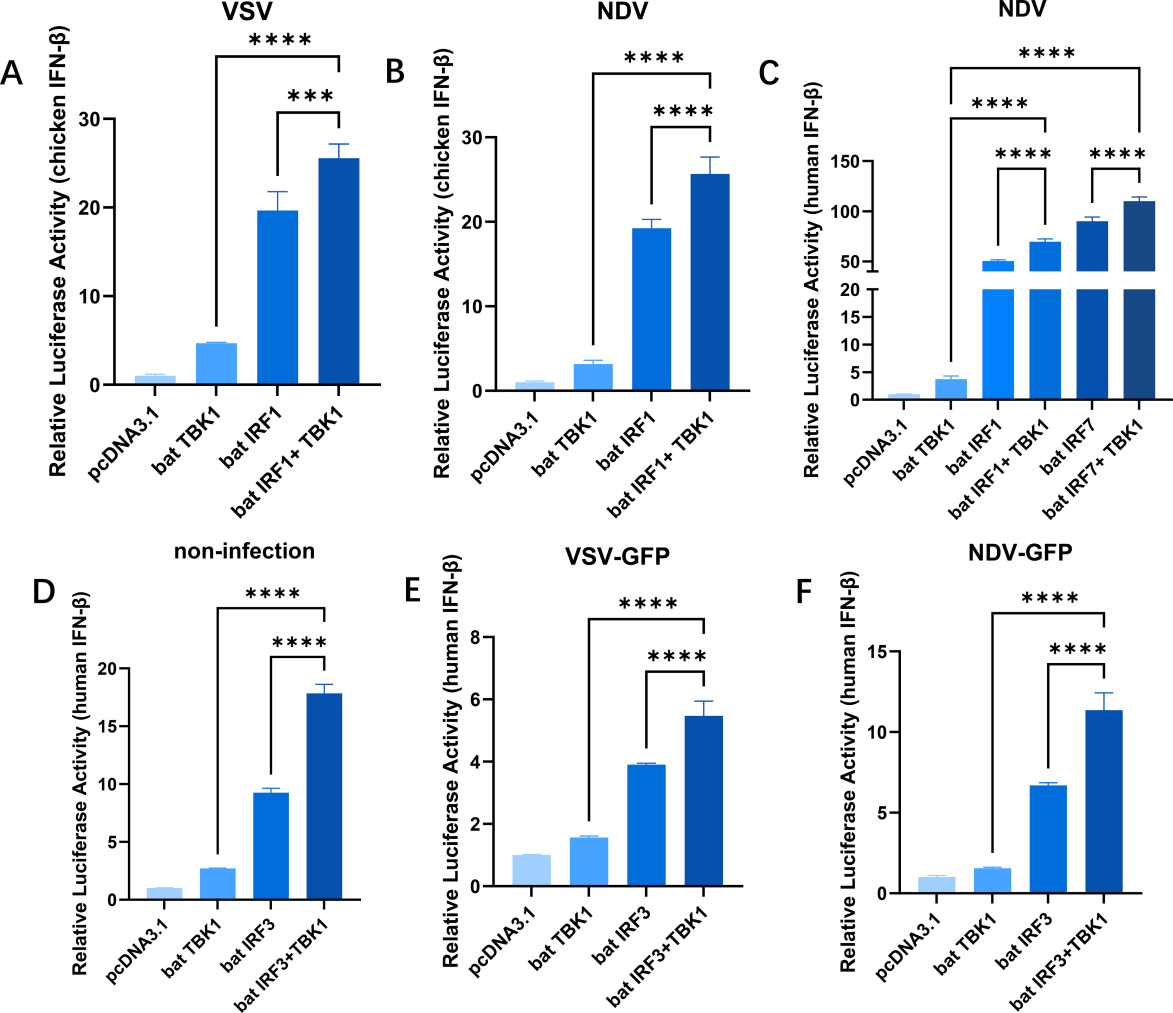
Bat TBK1 promotes IFNβ production through bat IRF1/3/7. **(A, B)** Overexpression of bat TBK1 facilitated the upregulation of IRF1-mediated IFNβ expression. DF1 cells were transfected with 500 ng of pcDNA3.1 alone, or co-transfected with pcDNA3.1(250 ng) and pcDNA3.1-bat-TBK1(250 ng), or pcDNA3.1(250 ng) and pcDNA3.1-bat-IRF1(250 ng), or pcDNA3.1-bat-TBK1(250 ng) and pcDNA3.1-bat-IRF1(250 ng). Six hours post-transfection, the cells were infected with VSV or NDV at 0.1 MOI. After 18 hours of viral infection, the cells were lysed, and dual-luciferase reporter assays were performed to measure IFNβ expression levels. **(C)** Overexpression of bat TBK1 facilitated the upregulation of IRF1/IRF7-mediated IFNβ expression. 293T cells were transfected with either 500 ng of pcDNA3.1 alone, or co-transfected with 250 ng of pcDNA3.1 and 250 ng of pcDNA3.1-bat-TBK1, or 250 ng of pcDNA3.1 and 250 ng of pcDNA3.1-bat-IRF1, or 250 ng of pcDNA3.1 and 250 ng of pcDNA3.1-bat-IRF7, or 250 ng of pcDNA3.1-bat-TBK1 and 250 ng of pcDNA3.1-bat-IRF1, or 250 ng of pcDNA3.1-bat-TBK1 and 250 ng of pcDNA3.1-bat-IRF7. Six hours post-transfection, the cells were infected with NDV at 0.1 MOI. After 18 hours of viral infection, the cells were lysed, and dual-luciferase reporter assays were performed to measure IFNβ expression levels. **(D–F)** Overexpression of bat TBK1 enhanced IRF3-mediated upregulation of IFNB expression. 293T cells were co-transfected with 120ng of PGL-human IFNβ-Luc, 60ng of PRL-TK and pcDNA3.1, or pcDNA3.1-bat-TBK1, or pcDNA3.1-bat-IRF3, or co-transfected with pcDNA3.1-bat-TBK1 and pcDNA3.1-bat-IRF3 (Each plasmid was transfected at 250 ng/well with the total amount of transfected plasmids maintained at 680 ng. The remaining portion was supplemented by empty pcDNA3.1 vector). Six hours post-transfection, the cells were infected with VSV or NDV at 0.1 MOI. After 18 hours of viral infection, the cells were lysed, and dual-luciferase reporter assays were performed to measure IFNβ expression levels. In this figure, * indicates P<0.05, ** indicates P<0.01, *** indicates P<0.001, **** indicates P<0.0001, ns indicates “not significant” (p > 0.05), all data were analyzed using one-way ANOVA, with normalization performed relative to the first column as the reference.

Previous studies have discovered that the bat TBK1 has a conserved function in promoting IFNβ expression and inhibiting viral replication. IRFs, especially IRF3/7, are important intermediate molecules in the process of TBK1 regulating IFNβ production. To clarify how bat TBK1 regulate IFNβ production, we conducted a study on IRFs. pcDNA3.1-bat-TBK1 and pcDNA3.1-bat-IRF1 were co-transfected into cells, upon VSV and NDV stimulation, overexpression of bat TBK1 facilitated the upregulation of IRF1-mediated IFNβ expression (**Figures 4A, B**). Similarly, under NDV stimulation, overexpression of bat TBK1 also enhanced the activation of downstream pathways mediated by IRF7 (**Figure 4C**). Furthermore, under the following conditions: without viral infection, during VSV infection, and during NDV infection, overexpression of bat TBK1 all enhanced IRF3-mediated upregulation of IFNβ expression. This suggests the activation signals potentially can be transmitted from bat TBK1 to IRF1/3/7, and then promote IFNβ production.

### 3.5 Essential domains of bat TBK1

**Figure 5:**
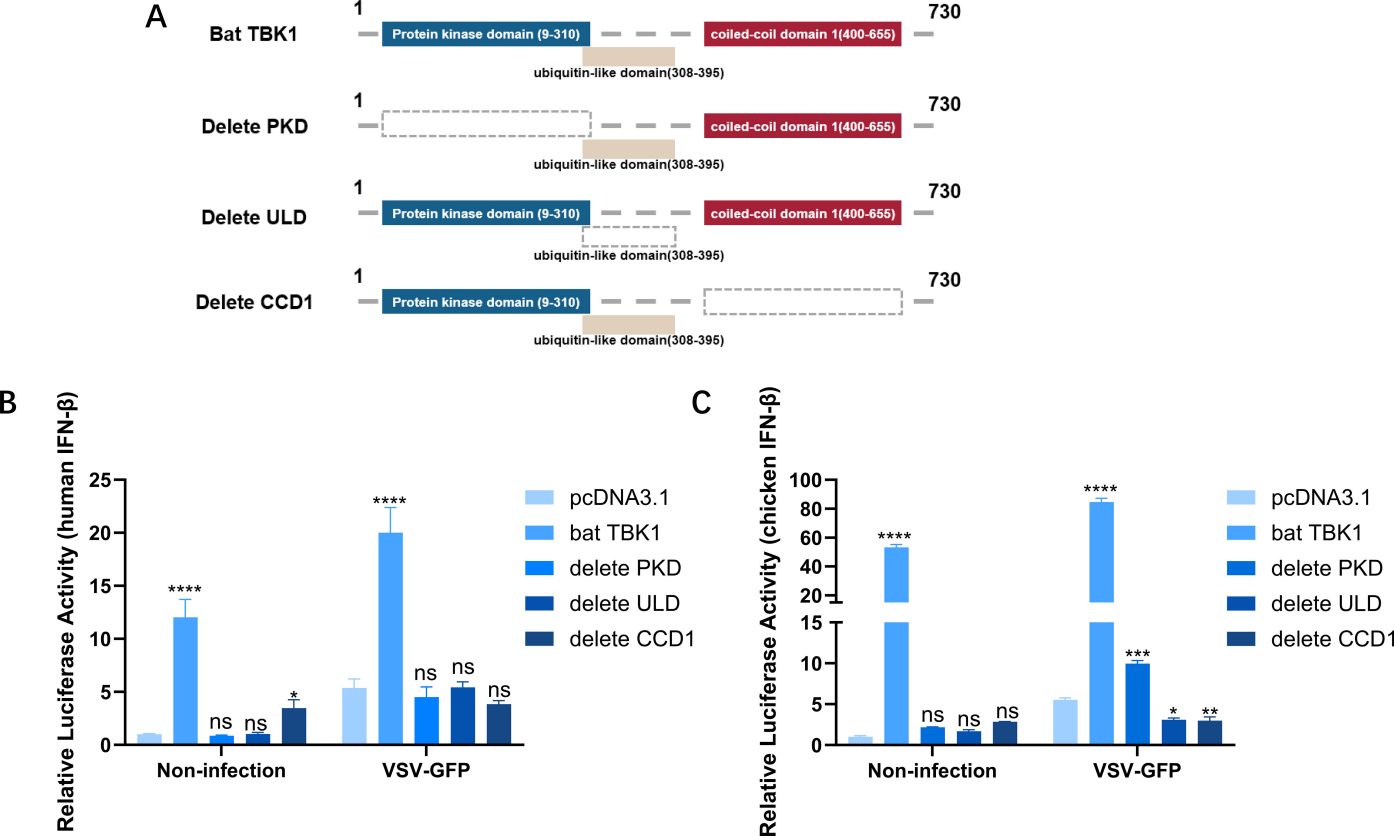
Essential domains of bat TBK1. **(A)** Schematic structure of bat TBK1 mutants. The deletion mutant plasmids of bat TBK1, namely pcDNA3.1-bat-TBK1-dPKD, pcDNA3.1-bat-TBK1-dULD, and pcDNA3.1-bat-TBK1-dCCD1, which lack the PKD, ULD, or CCD1 domains, respectively, were constructed by inverse PCR using the pcDNA3.1-bat-TBK1 plasmid as a template. **(B, C)** 250 ng of either pcDNA3.1, pcDNA3.1-bat-TBK1, pcDNA3.1-bat-TBK1-dPKD, pcDNA3.1-bat-TBK1-dULD, or pcDNA3.1-bat-TBK1-dCCD1 was co-transfected with 120 ng of PGL-IFNβ-Luc and 60 ng of PRL-TK into 293T or DF1 cells. Six hours post-transfection, the cells were infected with VSV at 0.1 MOI or non-infected. After 18 hours of viral infection, the cells were lysed, and dual-luciferase reporter assays were performed to measure IFNβ expression levels. Full-length bat TBK1 can upregulate IFNβ expression, while bat TBK1 deletion of the PKD, ULD, or CCD1 domains could only marginally or completely failed to upregulate IFNβ production. The data were analyzed using one-way ANOVA, with normalization performed relative to the first column as the reference. In this figure, * indicates P<0.05, ** indicates P<0.01, *** indicates P<0.001, **** indicates P<0.0001, ns indicates “not significant” (p > 0.05).

Structurally, bat TBK1 comprises a protein kinase domain (PKD: AA9-310), a ubiquitin-like domain (ULD: AA308-395), and coiled-coil domain 1 (CCD1: AA400-655). The deletion mutant plasmids of bat TBK1 were constructed by inverse PCR using the pcDNA3.1-bat-TBK1 plasmid as a template. The deleted domains are represented by grey dashed boxes. (**Figure 5A**). Overexpression of full-length TBK1 or TBK1 with deletions of the PKD, ULD, or CCD1 domains, respectively, full-length bat TBK1 can upregulate IFNβ expression, while bat TBK1 deletion of the PKD, ULD, or CCD1 domains could only marginally or completely failed to upregulate IFNβ production (**Figures 5B, C**), which underscores that the domains PKD, ULD, and CCD1 are indispensable for bat TBK1 to exert normal function in antiviral innate immunity.

## 4 Discussion

During millennia of evolution, bats have developed a myriad of distinctive traits, such as the ability to fly, employ laryngeal echolocation, and coexist with various viruses asymptomatically. Their unique high viral tolerance renders them excellent model organisms for studying antiviral drugs and therapies. However, limited understanding of bat immune systems currently hampers their research. Upon viral invasion, the innate immune system identifies the intrusion and upregulates inflammatory factors and type I interferon expression through the innate immune signaling network, thus controlling viral replication. Currently, research into the bat innate immune system remains in its infancy. Although recent studies have elucidated the functional characteristics of some bat innate immune signaling molecules including STING (17), MDA5 (18) and IRF1 (19). However, the functions of many crucial factors in the bat innate immune signaling network remain unclear, with TBK1 being one of them.

In this study, through bioinformatics analysis, TBK1 was found to exhibit a high sequence conservation across species. After VSV infection, TBK1 expression level was up-regulated. This suggests that bat TBK1 may possess a conserved antiviral innate immune function. Further research confirms this. Overexpression of bat TBK1 upregulates the expression of IFNβ, thereby inhibiting viral replication. This indicates the resemblance between bat TBK1 and its counterparts in other mammals, possessing a conserved function in regulating IFN production (20). Despite bats’ unique immune tolerance (14, 21), their TBK1 retains core innate immune signaling capacity, indicating this molecule plays an indispensable role in fundamental antiviral defense pathways. In addition we conducted preliminary exploration into the mechanism by which bat TBK1 regulates IFNβ production. IRFs are important intermediate molecules in the process of TBK1 regulating IFNβ production. Co-expression of bat TBK1 with bat IRF1/3/7 revealed that bat TBK1 can facilitate the upregulation of IRF1/IRF3/IRF7-mediated IFNβ expression, implying bat TBK1 posesses the ability to transmit activation signals to IRFs (IRF1/3/7). This is consistent with studies in other mammals, activated human TBK1 phosphorylates IRF3/7 leading their dimerization and translocation to the nucleus, where they drive the expression of antiviral type I (22, 23). What calls for special attention is that bat TBK1 constitutively enhances the IRF3-mediated interferon pathway under basal conditions (in the absence of viral infection; **Figure 4D**). This unique property may reflect an evolutionary adaptation allowing bats to maintain tonic immune activation at low levels, enabling rapid antiviral responses. Future studies should investigate whether species-specific regulatory mechanisms (e.g., post-translational modifications or interacting partners) govern bat TBK1’s activity. Structurally, the domains PKD, ULD, and CCD1 are indispensable for bat TBK1 to exert normal function in antiviral innate immunity. In humans, PKD mediates the serine phosphorylation function of TBK1 (24). It phosphorylates IRF3/IRF7, leading to their dimerization and nuclear translocation, where they induce IFN-α/β gene expression. IFN-α/β then trigger the expression of hundreds of interferon-stimulated genes (ISGs) that establish an antiviral state in neighboring cells. PKD also phosphorylates STAT proteins, enhancing antiviral signaling. ULD functions as a regulatory component involved in kinase activation, substrate delivery, and downstream signal pathway modulation (25). It helps in the oligomerization of TBK1, which is necessary for its autophosphorylation and full activation. Moreover, it contributes to the recruitment of adaptor proteins (e.g., MAVS, STING, or TRAF3) that are essential for downstream signaling. Mutations in the ULD can impair TBK1’s ability to activate IRF3, reducing IFN production and weakening antiviral responses. The CCD1 allows TBK1 to interact with TANK, NAP1, or SINTBAD, which are adaptor proteins that help recruit TBK1 to signaling platforms. It facilitates the formation of signalosomes. Without CCD1, TBK1 may fail to properly localize to key signaling hubs, reducing its efficiency in activating IRF3/IRF7. The high conservation of TBK1 sequences and the outcomes of TBK1 domain deletion experiments suggest potential functional similarities in domains between bat TBK1 and human TBK1.

In summary, this study, across multiple species-derived cell systems, including bat (TB1LU), mammalian (293T), and avian (DF1) cell lines, demonstrates that bat TBK1 possesses a conserved antiviral innate immune function: it activates IFNβ expression through IRF1/3/7 and inhibits NDV replication, with the PKD, ULD, and CCD domains being essential for its normal function. This research establishes a theoretical foundation for further elucidating the mechanisms of bat innate immunity. The study still has some limitations, for example, the functional mechanisms of bat TBK1 domains (PKD, ULD, and CCD1) remain to be fully elucidated, whether bat TBK1’s functional adaptations underlie bats’ exceptional viral reservoir status requires systematic investigation. Future studies can focus on these points and elucidate their mechanisms in detail.

## Data Availability

The original contributions presented in the study are included in the article/**Supplementary Material**. Further inquiries can be directed to the corresponding authors.
