# Supplementary material for: Conserved function of bat TBK1 in activating innate immunity against RNA viruses: insights into the innate immune response in bats
Source: Front Immunol. 2025 Jul 28;16:1574866. doi: 10.3389/fimmu.2025.1574866 (PMC12336183; doi:10.3389/fimmu.2025.1574866)
Supplement: Supplementary file 1 [file Table1.docx]

**Supplementary Table 1:**

Primers used in this study

| **Genes** | **Primer** | **Nucleotide sequence of primers (5’-3’)** |
| --- | --- | --- |
| BatTBK1 | Forward | ATGCAGAGCACTTCTAATC |
|  | Reverse | CTAAAGACAGTCAACATTGCG |
| pcDNA3.1 | Forward | CTCGAGGACTACAAGGACGACGAT |
|  | Reverse | GAATTCCACCACACTGGACTAGT |
| pcDNA3.1-batTBK1 | Forward | CCAGTGTGGTGGAATTCATGCAGAGCACTTCT |
|  | Reverse | CTTGTAGTCCTCGAGAAGACAGTCAACATTGC |
| BatTBK1-dPKD | Forward | ATTCATGTTTTTTCGCTACAACAAATGACAGCTC |
|  | Reverse | GTAGCGAAAAAACATGAATCAGATGATTAGAAGTGCTCTG |
| BatTBK1-dULD | Forward | AAAATTTCCCTCCCTAAAGTACACCCACGT |
|  | Reverse | AGGGAGGGAAATTTTGTGAAGTATATCACTAGTTTCTGC |
| batIRF7-dCCD1 | Forward | GAAAAAATTTCCCTCGAAACTTTGCCTCAG |
|  | Reverse | GAGGGAAATTTTTTCATACATCAATCCTATGG |
| qPCR-batIFN-β | Forward | TGCACTCTCCAGATGGCT |
|  | Reverse | CCTGCTCTCCATGCAATGT |
| qPCR-NDV | Forward | TGCAGCAATGGTACTCCGTT |
|  | Reverse | CCTTTGCTACCGTGACCCAT |
| qPCR-batTBK1 | Forward | ATGAAGGACGACGCCTAGTG |
|  | Reverse | GTCACAGGTTCCCGGCTTAC |

| **Genes** | **Primer** | **Nucleotide sequence of primers (5’-3’)** |
| --- | --- | --- |
| qPCR-batOAS1 | Forward | ATCTGCAGTTTCCTGAAGGAG |
|  | Reverse | GCTGAGGAAGCGACGAGGTC |
| qPCR-batIL6 | Forward | CTACTGCTTTCCCTACCC |
|  | Reverse | TCCTTGCTGTTTTTACACG |
| qPCR-batMX1 | Forward | GGAGGGTCAGCTCCCCTCA |
|  | Reverse | GCCATGCTCAGCGCCTCT |
| qPCR-batPKR | Forward | GGATTGCCCAAAAGGCAA |
|  | Reverse | GGTAGGACCTGTAGCATCA |
| qPCR-VSV(G gene) | Forward | TGCAAGGAAAGCATTGAACAA |
|  | Reverse | GAGGAGTCACCTGGACAATCAC |
| qPCR-chicken IFN-β | Forward | CCTCAACCAGATCCAGCATT |
|  | Reverse | GGATGAGGCTGTGAGAGGAG |
