# Supplementary material for: Conserved function of bat TBK1 in activating innate immunity against RNA viruses: insights into the innate immune response in bats
Source: Front Immunol. 2025 Jul 28;16:1574866. doi: 10.3389/fimmu.2025.1574866 (PMC12336183; doi:10.3389/fimmu.2025.1574866)
Supplement: Supplementary file 2 [file DataSheet1.pdf]

```
           1        10        20        30        40        50        60        70        80        90
bat        MQSTSNHLWLLSDILGQGATANVFRGRHKKTGDLFAIKVFNNISFLRPVDVQMREFEVLKKLNHKNIVKLFAIEEETTRHKVLIMEFCPCG
human      MQSTSNHLWLLSDILGQGATANVFRGRHKKTGDLFAIKVFNNISFLRPVDVQMREFEVLKKLNHKNIVKLFAIEEETTRHKVLIMEFCPCG
rabbit     MQSTSNHLWLLSDILGQGATANVFRGRHKKTGDLFAIKVFNNISFLRPVDVQMREFEVLKKLNHKNIVKLFAIEEETTRHKVLIMEFCPCG
pig        MQSTSNHLWLLSDILGQGATANVFRGRHKKTGDLFAIKVFNNISFLRPVDVQMREFEVLKKLNHKNIVKLFAIEEETTRHKVLIMEFCPCG
cow        MQSTSNHLWLLSDILGQGATANVFRGRHKKTGDLFAIKVFNNISFLRPVDVQMREFEVLKKLNHKNIVKLFAIEEETTRHKVLIMEFCPCG
horse      MQSTSNHLWLLSDILGQGATANVFRGRHKKTGDLFAIKVFNNISFLRPVDVQMREFEVLKKLNHKNIVKLFAIEEETTRHKVLVMEFCPCG
mouse      MQSTSNHLWLLSDILGQGATANVFRGRHKKTGDLYAVKVFNNISFLRPVDVQMREFEVLKKLNHKNIVKLFAIEEETTRHKVLIMEFCPCG
chicken    MQSTSNYLWLLSDILGQGATANVFRGRHKKTGDLYAVKVFNSISFLRPVDVQMREFEVLTKLNHKNIVKLFAIEEETTRHKVLVMEFCPCG
zebrafish  MQSTANYLWMMSDLLGQGATANVYRGRHKKTGDLYAVKVFNNLSFLRPLDVQMREFEVLKKLNHKNIVKLFAVEEESNTRHKVLVMEYCPCG

                   100       110       120       130       140       150       160       170       180
bat        SLYTVLEEPSNAYGLPESEFLIVLRDVVGGMNHLRENGIVHRDIKPGNIMRVIGEDGQSVYKLTDFGAARELEDDEQFVSLYGTEEYLHPDM
human      SLYTVLEEPSNAYGLPESEFLIVLRDVVGGMNHLRENGIVHRDIKPGNIMRVIGEDGQSVYKLTDFGAARELEDDEQFVSLYGTEEYLHPDM
rabbit     SLYTVLEEPSNAYGLPESEFLIVLRDVVGGMNHLRENGIVHRDIKPGNIMRVIGEDGQSVYKLTDFGAARELEDDEQFVSLYGTEEYLHPDM
pig        SLYTVLEEPSNAYGLPESEFLIVLRDVVGGMNHLRENGIVHRDIKPGNIMRVIGEDGQSVYKLTDFGAARELEDDEQFVSLYGTEEYLHPDM
cow        SLYTVLEEPSNAYGLPESEFLIVLRDVVGGMNHLRENGIVHRDIKPGNIMRVIGEDGQSVYKLTDFGAARELEDDEQFVSLYGTEEYLHPDM
horse      SLYTVLEEPSNAYGLPESEFLIVLRDVVGGMNHLRENGIVHRDIKPGNIMRVIGEDGQSVYKLTDFGAARELEDDEQFVSLYGTEEYLHPDM
mouse      SLYTVLEEPSNAYGLPESEFLIVLRDVVGGMNHLRENGIVHRDIKPGNIMRVIGEDGQSVYKLTDFGAARELEDDEQFVSLYGTEEYLHPDM
chicken    SLYTVLEEPSNAFGLPESEFLIVLRDVVAGMNHLRENGIVHRDIKPGNIMRVIGEDGQSVYKLTDFGAARELEDDEQFVSLYGTEEYLHPDM
zebrafish  SLYTVLEEPTNAYGLPEDEFLIVLQDVVAGMNHLREYGIVHRDIKPGNIMRVIGDDGFSVYKLTDFGAARELEDDEQFVSLYGTEEYLHPDM

                190       200       210       220       230       240       250       260       270
bat        YERAVLRKDHQKKYGATVDLWSIGVTFYHAATGSLPFRPFEGPRRNKEVMYKIITGKPSGAISGVQKAENGPIDWSGDMPVSCSLSRGLQVL
human      YERAVLRKDHQKKYGATVDLWSIGVTFYHAATGSLPFRPFEGPRRNKEVMYKIITGKPSGAISGVQKAENGPIDWSGDMPVSCSLSRGLQVL
rabbit     YERAVLRKDHQKKYGATVDLWSIGVTFYHAATGSLPFRPFEGPRRNKEVMYKIITGKPSGAISGVQKAENGPIDWSADMPVSCSLSRGLQVL
pig        YERAVLRKDHQKKYGATVDLWSIGVTFYHAATGSLPFRPFEGPRRNKEVMYKIITGKPSGAISGVQKAENGPIDWSGDMPVSCSLSRGLQVL
cow        YERAVLRKDHQKKYGATVDLWSIGVTFYHAATGSLPFRPFEGPRRNKEVMYKIITGKPSGAISGVQKAENGPIDWSGDMPVSCSLSRGLQVL
horse      YERAVLRKDHQKKYGATVDLWSIGVTFYHAATGSLPFRPFEGPRRNKEVMYKILTGKPSGAISGVQKAENGPIDWSGDMPVSCSLSRGLQVL
mouse      YERAVLRKDHQKKYGATVDLWSVGVTFYHAATGSLPFRPFEGPRRNKEVMYKIITGKPSGAISGVQKAENGPIDWSGDMPLSCSLSQGLQAL
chicken    YERAVLRKEHQKKYGATVDLWSIGVTFYHAATGSLPFRPFEGPRRNKEVMYKIITGKPSGAISGIQKAANGPIEWSWEMPISCSLSKGLQVL
zebrafish  YERAVLRKDHQKKYGATVDLWSIGVTFYHAATGSLPFRPFEGPRRNKEVMYKIITEKPPGAISGHQKFENGKIEWSSEMPISCSLSKGLQSL

              280       290       300       310       320       330       340       350       360
bat        LTPVLANILEADQEKCWGFDQFFAETSDILHRMIIHVFSLQQMTAHKIYIHSYNTATVFHELVYKQTKIISANQQLIYEGRRLVLEPGRLVQ
human      LTPVLANILEADQEKCWGFDQFFAETSDILHRMVIHVFSLQQMTAHKIYIHSYNTATIFHELVYKQTKIISSNQELIYEGRRLVLEPGRLAQ
rabbit     LTPVLANILEADQEKCWGFDQFFAETSDILHRMIIHVFSLQQMTAHKIYIHSYNTATVFHELVYKQTKIISSNQELIYEGRRLVLEPGRLAQ
pig        LTPVLANILEADQEKCWGFDQFFAETSDILHRMIIHVFSLQQMTAHKIYIHSYNTATVFHELVYKQTKIISSNQELIYEGRRLVLEPGRLAQ
cow        LTPVLANILEADQEKCWGFDQFFAETSDILHRMIVHVFSLQQMTAHKVYIHGYNTATVFHELVYKQTKIISSNQELIYEGRRLVLEPGRLAQ
horse      LTPVLANILEADQEKCWGFDQFFAETSDILHRMIIHVFSLQQMTAHKIYIHSYNTAAVFHELVYKQTKIISSNQELIYEGRRLVLEPGRLAQ
mouse      LTPVLANILEADQEKCWGFDQFFAETSDVLHRMVIHVFSLQHMTAHKIYIHSYNTAAVFHELVYKQTKIVSSNQELIYEGRRLVLELGRLAQ
chicken    LTPVLANILEADQEKCWGFDQFFAETSDILHRIIIHIFSLQQMTLHKVYIHSYNTAAIFHELVYKQTKIPSQNQELIYEGRRLILEPGRLAQ
zebrafish  LTPVLANILEADQEKCWGFDQFFAETSDILHRIVVYVFSLQQATLHHVYIHTYNTANLFQELLFRRTNITPSHQELLYEGRRLVLDPNRQAQ

          370       380       390       400       410       420       430       440       450       460
bat        HFPKTTEENPIFIVSREPVTTIGLMYEKISLPKVHPRYDLDGDASMAKAITGVVCYACRIASTLLLYQELMRKGIRWLIELVKDDYNETVHK
human      HFPKTTEENPIFVVSREPLNTIGLIYEKISLPKVHPRYDLDGDASMAKAITGVVCYACRIASTLLLYQELMRKGIRWLIELIKDDYNETVHK
rabbit     HFPKTTEENPIFVVSREPLSTIGLIYEKISLPKVHPRYDLDGDASMAKAITGVVCYACRIAGTLLLYQELMRKGIRWLIELVKDDYNEAVHK
pig        HFPETTEENPIFVVSREPLSTIGLIYEKISLPKVHPRYDLDGDASVAKAITGIVCYACRIASTLLLYQELMRKGIRWLIELVKDDYNETVHK
cow        HFPKTSEENPIFVVSREPLSTIGLIYEKISLPKVHPRYDLDGDASMAKAMTGVVCYACRIASTLLLYQELVRKGIRWLIELVKDDYNETVHK
horse      HLPKTTEDNPIFVVSQEPLSAVGLIYEKISLPKVHPRYDLDGDASMAKAITGVVCYACRVTSTLLLYQELMRKGIRWLIELVKDDYNETVHK
mouse      HFPKTTEENPIFVTSREQLNTVGLRYEKISLPKIHPRYDLDGDASMAKAVTGVVCYACRTASTLLLYQELMRKGVRWLVELVKDDYNETVHK
chicken    HFPRTTEENPIFVVSREAVNIVGLVYEEVLLPKVHQRYDLDGDASMAKVITRIVCYACRVANSLLLYQELMRRGIRWLIEIIKDDYNEMVHK
zebrafish  TFPKTSRDNPIMLLCRDPVNTVGLLFEDPSPPKVQPRYDLDLDASYAKTFAGDVGYLWKTSDSLLLYQELVRKGVRGLNELIRDEYSETMHK

                   470       480       490       500       510       520       530       540       550
bat        KTEVVITLDFCIRNIEKTVNVYEKLMKINLEAAELDEISDIHTKLLRLSSSQGTIETSLQDIESKLSPGGLLSDTWAHQEGTHPKDRHVEKL
human      KTEVVITLDFCIRNIEKTVKVYEKLMKINLEAAELGEISDIHTKLLRLSSSQGTIETSLQDIDSRLSPGGSLADAWAHQEGTHPKDRNVEKL
rabbit     KTEVVITLDFCIRNIEKTVKVYEKLMKINLEAAELGEISDIHTKLLRLSSSQGTIETSLQDVDSRLSPGGLLADAWAHQEGTHPKDRNVEKL
pig        KTEVVITLDFYIRNIEKTVTVYEKLMKINLETTELGEISDIHTKLLRLSSSQGTIETSLQDIESKLSPGGLLADTWTHQEGTHPKDRHVEKL
cow        KTEVVITLDFCIRNIEKTVKVYEKLMKINLEAAELGEISDIHTKLLRLSSSQGTIETSLQDIESKLSPGGLLADTWTHQEGTHPKDRHVEKL
horse      KTEVVITLDFCIRNIEKTMKVYEKLIEINLEAAELGEISDIHTKLLRLSSSQGTIETSLQDIESKLSPSGLLTDAWAHQEGTHPKDRHVEKL
mouse      KTEVVITLDFCIRNIEKTVKVYEKLMKVNLEAAELGEISDIHTKLLRLSSSQGTIESSLQDISSRLSPGGLLADTWAHQEGTHPKDRNVEKL
chicken    KTEVVIRLDFCSRNIEKAEKIYENLMQINLESSEVDEISEINTKLLRLSSSQGTIETSLQDIKTKLSPGGLLADTWANQEGTHPRDRNSERL
zebrafish  KTEVFHLCSHCSQTLERSEQLCEALMQGNILSAEYDEIRDTRKKVLRLSGSLASMDQTLQDINSMFLPGGSLTDTWTQQVGTHPEDRNVEKI

                560       570       580       590       600       610       620       630       640
bat        QVLLNCITEIYYQFKKDKAERRLAYNEEQIHKFDKQKLYYHATKAMTHFTDECVQKYETFLDKSEDWMRKMLHLRKQLLSLTNQCFDIEEEV
human      QVLLNCMTEIYYQFKKDKAERRLAYNEEQIHKFDKQKLYYHATKAMTHFTDECVKKYEAFLNKSEEWIRKMLHLRKQLLSLTNQCFDIEEEV
rabbit     QVLLNCITEIYYQFKKDKAERRLAYNEEQIHKFDKQKLYYHATKAMTHFSDECVKKYEAFLDKSEEWMRKMLHLRKQLLSLTNQCFDIEEEV
pig        QVLLNCITEIYYQFKKDKAERRLAYNEEQIHKFDKQKLFYHATKAMTHFTDECVKKYEAFLDKSEEWMRKMLHLRKQLLSLTNQCFDIEEEV
cow        QVLLNCITEIYYQFKKDKAERRLAYNEEQIHKFDKQKLYYHATKAMTHFTEECVKKYEAFLDKSEEWMRKMLHLRKQLLSLTNQCFDIEEEV
horse      QVLLSCITEIYYQFKKDKAERRLAYNEEQIHKFDKQKLYYHATKAMTHFTDECVKKYEVFLDKSEEWMRKMLHLRKQLLALTNQCFDIEEEV
mouse      QVLLNCITEIYYQFKKDKAERRLAYNEEQIHKFDKQKLYYHATKAMSHFSEECVRKYEAFKDKSEEWMRKMLHLRKQLLSLTNQCFDIEEEV
chicken    QTLLSAITDIYYQFKKDKQERRLAYNEEQIHKFDKQKLYLHATKAIALFKDECVSKYDAFLDKAEDWTRKMLHTRKQLLALTNQCFDIEEEV
zebrafish  KVLLDAIGAIYQQFKKDKAERRLPYNEEQIHKFDKQKLVLHATKARALFTDECAMKYRLFISKSEEWMKKFHHVRKHLLSLTGQFSSLEQEV

              650       660       670       680       690       700       710       720
bat        SKYQDYTNELQETLPQKMFAASSGIKHTMAPIYPCSNTLVEMTLGMKKLKEEMEGVVKELAENNHILERFGSLTMDGGLRNVDCL
human      SKYQEYTNELQETLPQKMFTASSGIKHTMTPIYPSSNTLVEMTLGMKKLKEEMEGVVKELAENNHILERFGSLTMDGGLRNVDCL
rabbit     SKYQDYTNELQETLPQKMFAASSGIKHTMTPVYPSSNTLVEMTLGMKKLKEEMEGVVKELAENNHILERFGSLTMDGGLRNVDCL
pig        SKYQDYTNELQETLPQKMFAASSGIKHTMTPIYPCSNTLVEMTLGMKKLKEEMEGVVKELAENNHILERFGSLTMDGGLRNVDCL
cow        SKYQEYTNELQETLPQKMFAASSGIKHTMTPIYPCSNTLVEMTLGMKKLKEEMEGVVKELAENNHILERFGSLTMDGGLRNVDCL
horse      SKYQDYTSELQETLPQKMFAASSGMKHAMTPVYPCSNTLVEMTLGMKKLKEEMEGVVKELAENNHILERFGALTMDGGLRNVDCL
mouse      SKYQDYTNELQETLPQKMLAASGGVKHAMAPIYPSSNTLVEMTLGMKKLKEEMEGVVKELAENNHILERFGSLTMDGGLRNVDCL
chicken    SKYQDYINELQDALPQKMFAASTGMKHTMNTVYPSSNTLVEMTLGMKKLKEEMEGVVKELAENNHILERFGALTADGGLRNVDCI
zebrafish  TLLMQRLYKLLEQFPQKVVPMASGVLKPQAYLSPS..TLVEMTLGMKKLKEEMEGVVKELAENNLFLERFGSLTVDGGMRTVERM
```
